# Finerenone attenuates myocardial apoptosis, metabolic disturbance and myocardial fibrosis in type 2 diabetes mellitus

**DOI:** 10.1186/s13098-023-01064-3

**Published:** 2023-04-29

**Authors:** Tao Jin, Xiangrui Fu, Ming Liu, Fengshuang An

**Affiliations:** The Key Laboratory of Cardiovascular Remodeling and Function Research, Chinese Ministry of Education, Chinese National Health Commission and Chinese Academy of Medical Sciences, The State and Shandong Province Joint Key Laboratory of Translational Cardiovascular Medicine, Department of Cardiology, Qilu Hospital of Shandong University, Jinan, China

**Keywords:** Diabetic cardiomyopathy, Finerenone, MRA, Fibrosis, Apoptosis, PPAR, CD36, TNFα

## Abstract

**Background:**

Finerenone is a third-generation mineralocorticoid receptor antagonists, which has shown good cardiac function improvement in patients with type 2 diabetes in large-scale clinical trials. However, its specific role in diabetic cardiomyopathy remains unclear. We explored the potential functions and mechanisms of finerenone in diabetic cardiomyopathy.

**Methods:**

The type 2 diabetic rat model was induced by high-fat diet and low-dose streptozotocin (n = 6, each group). Next the drug group was treated with finerenone (1 mg/kg/day) for 8 weeks. Then we detected the cardiac structure and function and relevant indicators. Neonatal rat cardiomyocytes were used for in vitro culture to determine the direct effect of finerenone on cardiomyocytes stimulated by high glucose and high fatty acid.

**Results:**

Compared with the control group, rats in the type 2 diabetes group exhibited hyperglycemia, hyperlipidemia, and impaired cardiac function. Myocardium showed increased fibrosis and apoptosis. Finerenone attenuated these impairments without changing blood glucose levels. In neonatal rat cardiomyocytes, the stimulation of high concentrations of palmitic acid increased fatty acid uptake, as well as increased reactive oxygen species and apoptosis. Finerenone significantly improved fatty acid metabolism, reduced cellular inflammation levels, and decreased apoptosis.

**Conclusions:**

By blocking the mineralocorticoid receptor, finerenone attenuates cardiac steatosis, myocardial fibrosis and apoptosis, and subsequent myocardial remodeling and diastolic dysfunction in type II diabetic rats.

**Supplementary Information:**

The online version contains supplementary material available at 10.1186/s13098-023-01064-3.

## Introduction

Diabetes is a growing global disease, with more than 500 million people suffering from it according to statistics [1]. Among these patients, even without vascular disease and other cardiac abnormalities, heart failure develops, called diabetic cardiomyopathy (DCM) [2]. The main features of DCM are abnormal myocardial lipid metabolism, apoptosis, fibrosis and myocardial remodeling [3]. However, patients with diabetes and obesity have significantly higher levels of myocardial fatty degeneration and are more prone to impaired cardiac function [4]. Excess free fatty acids (FFA) in the blood may lead to increased cardiac FFA uptake and increased intracellular fatty acid (FA), leading to lipotoxicity of downstream metabolites such as reactive oxygen species (ROS) [5]. Excessive activation of the renin–angiotensin–aldosterone-system (RAAS) can be seen in DCM [6], aldosterone levels in diabetic patients are higher than normal people [7], the activation of aldosterone can cause cardiomyocyte fibrosis [8], and aldosterone can also cause apoptosis through the mitochondrial pathway [9].

Finerenone is the third generation of mineralocorticoid receptor antagonists (MRAs). Compared with the previous MRAs, finerenone has great specificity for mineralocorticoid receptor (MR) [10], which gives finerenone great application potential. In large-scale clinical trials, finerenone has demonstrated strong cardiac benefits [11, 12], including reduced cardiovascular mortality and reduced cardiac fibrosis. However, the effect of finerenone on DCM and its related molecular mechanism, especially in steatosis and apoptosis, have not been reported yet. In this study, we analyzed the changes in the transcriptome level of cardiomyocytes under finerenone treatment, and studied the possible mechanism of finerenone’s protective effect through further cell and animal experiments.

## Materials and methods

### Animal model

Eighteen 8-week-old Sprague‐Dawley rats were randomly divided into three groups (n = 6). All rats were maintained on a light–dark cycle at 23 °C. The control group was fed a basic diet (12.79% fat, 20.54% protein, 66.67% carbohydrate), and the rest were fed a high-fat diet (47.18% fat, 19.54% protein, 33.29% carbohydrate). Then intraperitoneal glucose tolerance test (IPGTT) and intraperitoneal insulin tolerance test (IPITT) were performed to identify insulin‐resistant rats. A single intraperitoneal injection of streptozotocin (40 mg/kg) was performed on insulin-resistant rats after a high-fat diet to induce a type 2 diabetes model [13, 14]. Fasting blood glucose (FBG) was measured 7 days after injection, and rats with FBG ≥ 11.1 mmol/L were considered as successful diabetes models. After 8 weeks of hyperglycemia, the drug group was treated with finerenone (1 mg/kg/day). All rats were sacrificed after 8 weeks of finerenone treatment, and all experimental protocols were legally approved by the Animal Care Committee of Shandong University.

### Cardiac function

Cardiac function in rats was measured by the Vevo2100 imaging system with an MX250 probe (VisualSonics, Toronto, Canada). The echocardiography parameters involved the left ventricular ejection fraction (LVEF), peak E to peak A ratio (E/A), early (eʹ) to late (aʹ) diastolic velocity ratio (e/a) and the fractional shortening (FS).

### Histology staining

Hearts were fixed with 4% paraformaldehyde, embedded in paraffin, and cut into 4 μm sections for haematoxylin and eosin (HE) staining. We performed Masson’s trichrome to measure the level of fibrosis.

### Cell treatment

Neonatal rat cardiomyocytes (NRCMs) were extracted for cell culture, and the medium used was dulbecco’s modified eagle medium (DMEM) (5% horse serum, 8% calf serum, 0.1 mM 5-Bromodeoxyuridinc). Hyperlipidemia or hyperglycemia regulation was mimicked by incubation with high concentrations of common saturated FFA (sodium palmitate, 16:0, 0.3 mM sigma) or glucose (d-glucose, 33 mM). Palmitate was previously conjugated with Bovine Serum Albumin (BSA) in a 3:1 molar ratio as published elsewhere [15]. In control cells, BSA was added as described but in the absence of palmitate. Finerenone (20 nM) was added 1 h before stimulation.

### RNA-seq and transcriptome analysis

The total RNA from the NRCMs was extracted according to the instruction manual of the TRlZOl Reagent (Life technologies, California, USA). RNA concentration and purity was measured using NanoDrop 2000 (Thermo Fisher Scientific, Wilmington, DE). RNA integrity was assessed using the RNA Nano 6000 Assay Kit of the Agilent Bioanalyzer 2100 system (Agilent Technologies, CA, USA). After enrichment of mRNA, the cDNA library was constructed, followed by bulk RNA sequencing and transcriptome analysis on the Illumina Novaseq system, entrusted to Biomarker Technologies Inc. (Beijing, China). Differential expression analysis of two groups was performed using the DESeq2. DESeq2 provide statistical routines for determining differential expression in digital gene expression data using a model based on the negative binomial distribution. The resulting P values were adjusted using the Benjamini and Hochberg’s approach for controlling the false discovery rate. Genes with an adjusted P-value < 0.05 and Fold Change ≥ 1.5 found by DESeq2 were assigned as differentially expressed.

### Measurement of intracellular ROS

Oxidative stress was evaluated via detecting the production of intracellular ROS in cultured NRCMs after different treatments. Cells were incubated with 20 µM 2′,7′‐dichlorofluorescein‐diacetate (DCFH‐DA, Solarbio) for 15 min and then washed three times with phosphate‐buffered saline for removing residual DCFH probe. The fluorescence intensity was observed under an inverted fluorescence microscope (Nikon).

### Examination of apoptosis

TUNEL and Annexin V/PI staining were used to measure cell apoptosis [16, 17]. For DNA fragmentation, TUNEL assays kit (Roche, USA) was used to treat the NRCMs or tissue sections according to manufacturer’s instructions. Images were acquired via a fluorescence microscopy (Nikon). FITC Annexin V Apoptosis Detection Kit I (BD, USA) was used to determine the stage of cell apoptosis. Data were acquired by flow cytometry (FACSCalibur, BD, USA).

### Immunofluorescence microscopy

Immunofluorescence was used to visualize the expression and localization of target proteins. NRCMs were fixed with 4% paraformaldehyde, permeabilized, and then blocked with 2% BSA for 2 h. Thereafter, cells were incubated with mouse anti-TNFα (Proteintech, 60291-1-lg) and rabbit anti-TNFR1 (Proteintech, 21574-1-AP) antibodies overnight at 4 °C. After incubation with two different fluorescent secondary antibodies for 60 min at 37 °C and staining with DAPI, images were obtained by observation with a confocal fluorescence microscope (LSM710, CarlZeiss, Germany).

### Western blot

Cardiac tissue or cells were lysed with RIPA lysis buffer. The prepared protein samples were separated by 12% sodium dodecyl sulphate polyacrylamide gel electrophoresis (SDS-PAGE) and then transferred to PVDF membrane (Millipore, USA). 5% nonfat milk was used for blocking at room temperature for 1 h, and then the membrane was incubated with 1 antibody at 4 °C overnight. The next day, incubation with secondary antibodies was performed for 1 h at room temperature before enhanced chemiluminescence using an Amersham Imager 800 (Additional file 1).

### Statistical analysis

All analyzes were carried out in Prism 6.0 (Graphpad) and SPSS 20.0. One-way ANOVA was used to compare the differences between groups and unpaired t-test was used for the differences between two groups. Each experiment was repeated at least three times, and data are shown as mean ± standard deviation (SD). Two-tailed P < 0.05 was considered statistically significant.

## Results

### Basic characteristic of type 2 diabetic rats

Rats with type 2 diabetes mellitus (T2DM) showed higher levels of blood glucose, free fatty acids, and higher heart weight to body weight ratios. After 8 weeks of finerenone treatment, there was no significant difference in blood glucose and heart weight to body weight ratio, and blood lipids seemed to increase, but this difference did not reach statistical significance (Table 1). Renal function (urea nitrogen, creatinine, albumin) and liver function (aspartate and alanine aminotransferase) injury markers remained within normal ranges in all groups (not shown).

**Table 1:** Basic information of rats

	Control	DM	DM + Finerenone
TC (mmol/L)	1.78 ± 0.28	2.38 ± 0.32*	3.31 ± 1.51
HDL-C (mmol/L)	1.18 ± 0.21	0.98 ± 0.22	1.01 ± 0.39
LDL-C (mmol/L)	0.22 ± 0.06	0.75 ± 0.38*	1.24 ± 0.91
TG (mmol/L)	0.92 ± 0.31	3.29 ± 1.22*	4.57 ± 3.31
FFA (umol/dL)	25.88 ± 6.15	50.72 ± 12.16*	70.55 ± 25.88
GLU (mmol/L)	8.6 ± 1.67	23.47 ± 2.17*	25.07 ± 0.85
ALD (pg/mL)	246.45 ± 86.22	219.34 ± 47.17	262.95 ± 18.8
Weight(g)	651.33 ± 91.11	465.17 ± 54.84	483.83 ± 30.87
HW(g)	2.24 ± 0.27	2.08 ± 0.11	2.25 ± 0.3
HW/Weight (mg/g)	3.45 ± 0.23	4.5 ± 0.32*	4.63 ± 0.44

TC, total cholesterol; HDL-C, high-density lipoprotein cholesterol; LDL-C, low-density lipoprotein Cholesterol; TG, total triglyceride; FFA, free fatty acids; GLU, glucose; ALD, aldosterone; HW, heart weight

^*^P < 0.05 compared with the control group

### Finerenone attenuates cardiac hypertrophy and cardiac dysfunction in diabetic rats

**Fig. 1:**
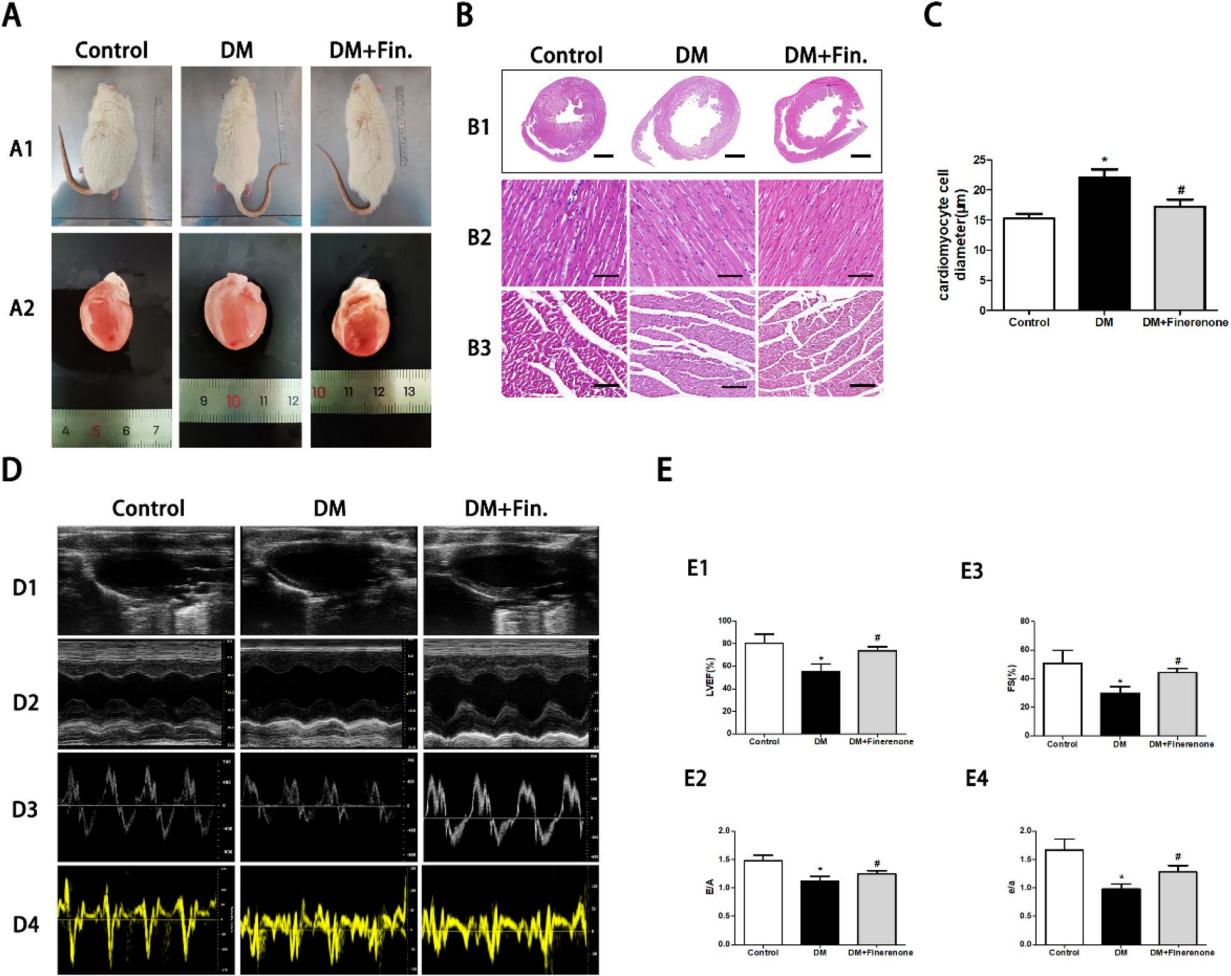
Finerenone attenuates left ventricular remodeling and improves cardiac function in diabetic rats. **A** General and heart morphology photos of rats. **B** HE staining of heart. **B1** HE staining of cross shaft of musculi papillares in heart. **B2** Representative HE staining of longitudinal left ventricular (LV) sections. **B3** Representative HE staining of LV transverse sections. **C** Quantitative analysis of the cardiomyocyte diameter in heart tissue. **D** Representative images of 2D echocardiograms (**D1**), M‐mode echocardiograms (**D2**), Pulse‐wave Doppler echocardiograms of mitral inflow (**D3**), tissue Doppler echocardiograms (**D4**). **E** Assessment of cardiac function, including left ventricular ejection fraction (LVEF), fractional shortening (FS), early to late mitral diastolic blood ratio (E/A), and diastolic mitral annular velocity ratio (E′/A′). *P < 0.05 compared with control; ^#^P < 0.05 compared with DM; Data are means ± SD

Compared with the control group, the hearts of diabetic rats showed the characteristics of pathological hypertrophy (Fig. 1A), and the diameter of cardiomyocytes was significantly increased (Fig. 1B, C). Finerenone treatment significantly reduced cardiomyocyte diameter in rats compared with the untreated group.

The heart function of diabetic rats was examined by ultrasound (Fig. 1D, E). Compared with the control group, the LVEF, F/S, E/A, Eʹ/Aʹ indexes of diabetic rats were abnormal, showing left ventricular systolic and diastolic dysfunction. Cardiac dysfunction was improved in finerenone-treated diabetic rats compared with the untreated group.

### Finerenone attenuates myocardial fibrosis and apoptosis in the heart of diabetic rats

**Fig. 2:**
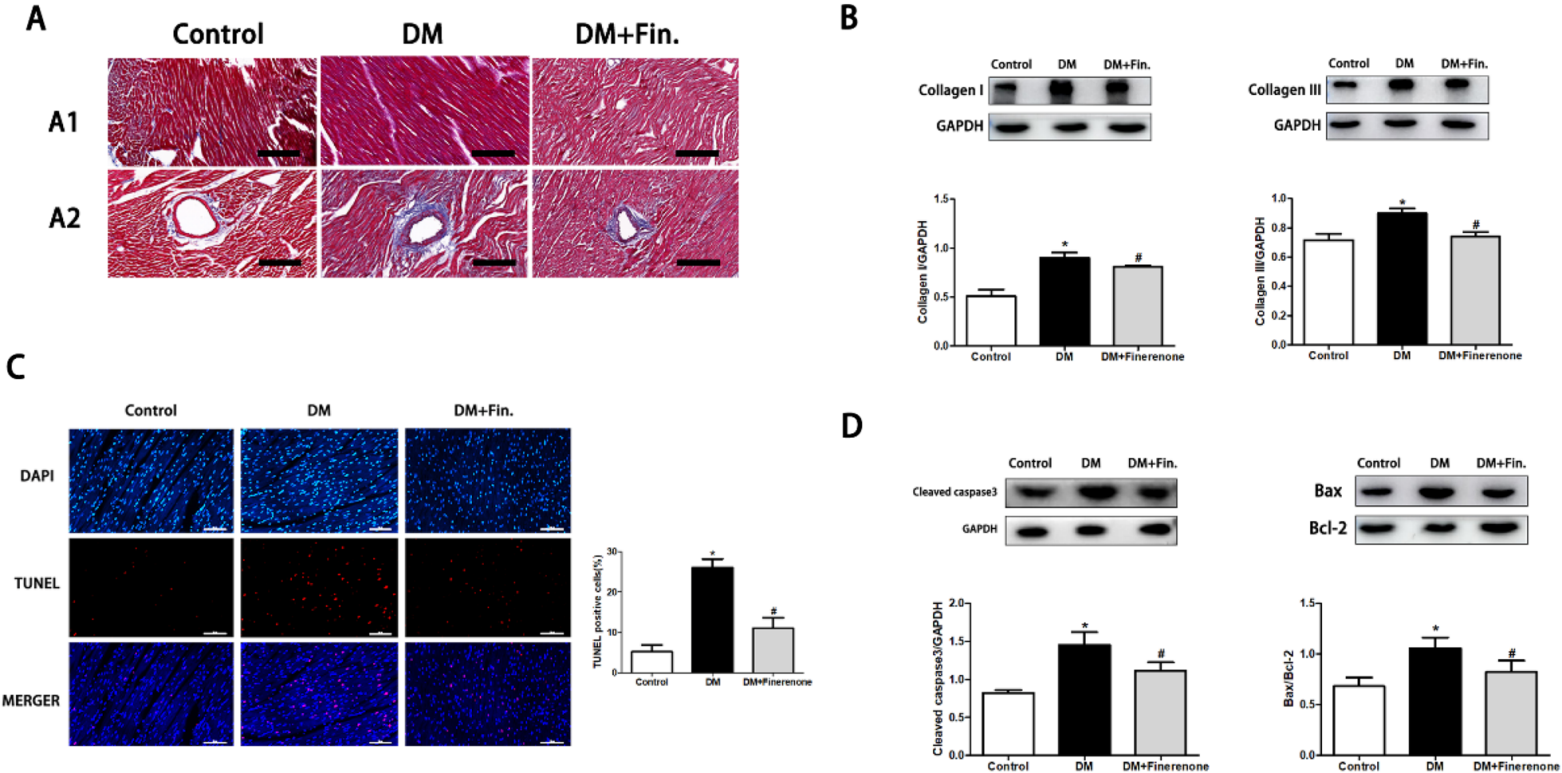
Finerenone attenuates myocardial fibrosis and apoptosis in the heart of diabetic rats. **A** Masson’s trichrome staining of myocardial mesenchyme and peripheral vessel. **B** Representative images of the Western blot of collagen I and collagen III, with the corresponding analysis. **C** Cell apoptosis as determined by TUNEL assay with the corresponding analysis: apoptosis cell stained red; nuclei stained blue with DAPI. **D** Representative images of the Western blot of Cleaved-Casp3, BAX and BCL-2 with the corresponding analysis. *P < 0.05 compared with control; ^#^P < 0.05 compared with DM; Data are means ± SD

Compared with the control group, Masson showed that the level of extracellular matrix in the interstitial area was increased in the diabetic group (Fig. 2A). The area of fibrosis among cardiomyocytes increased, and the expressions of collagen I and collagen III increased significantly (Fig. 2B). Myocardial fibrosis levels were reduced after finerenone treatment. At the same time, TUNEL showed that the apoptosis level of cardiomyocytes in the diabetic group increased (Fig. 2C), and the apoptosis marker proteins Cleaved-Caspase3 and BAX/BCL-2 both increased (Fig. 2D). After finerenone treatment, the level of myocardial apoptosis decreased.

### Finerenone reduces the apoptosis of cardiomyocytes stimulated by high glucose and high fatty acid

**Fig. 3:**
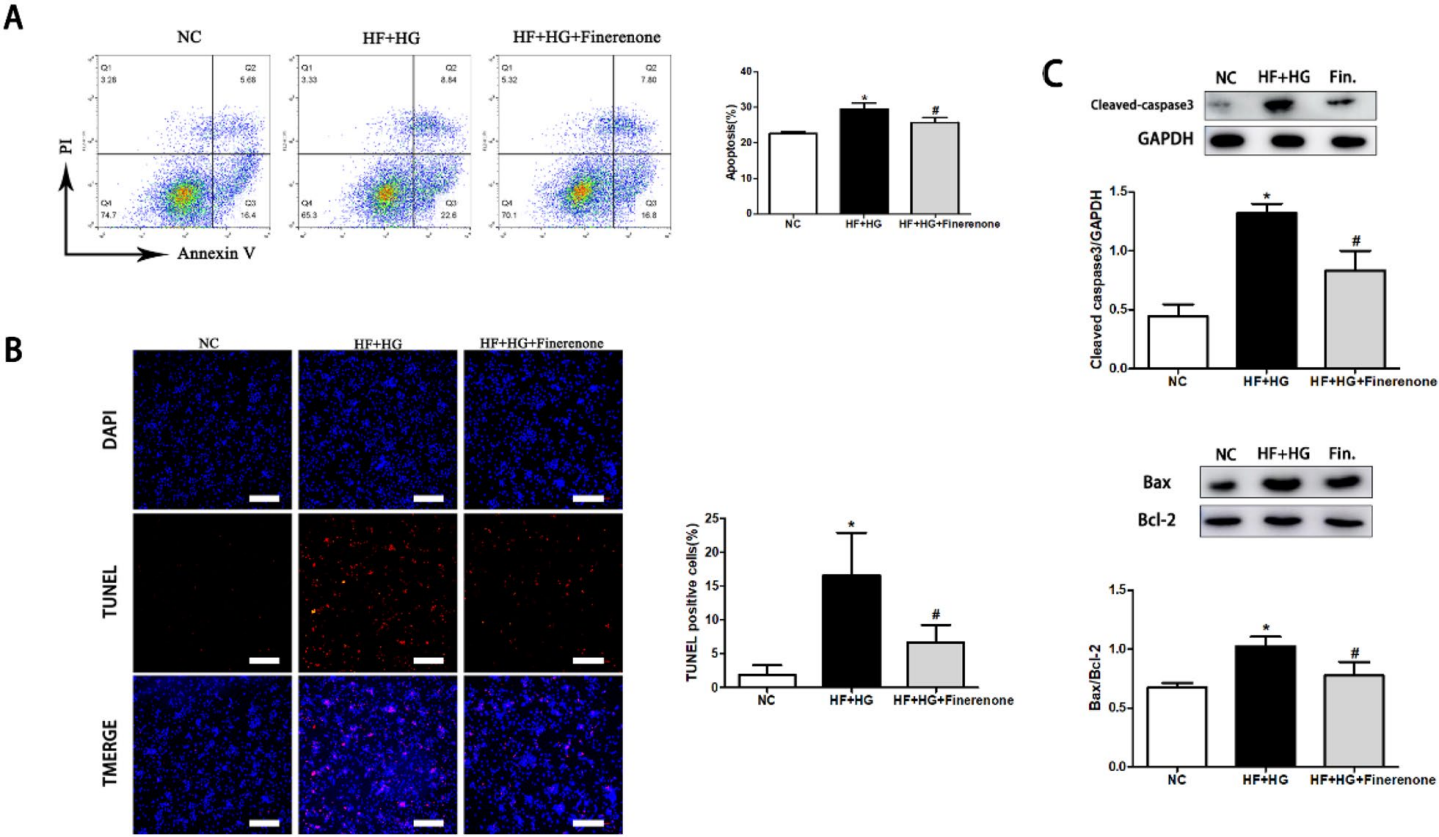
Finerenone attenuates apoptosis in neonatal rat myocardium. **A** Flow cytometry and quantification of apoptotic NRCMs with the corresponding analysis. **B** NRCMs apoptosis as determined by TUNEL assay with the corresponding analysis: apoptosis cell stained red; nuclei stained blue with DAPI. **C** Representative images of the Western blot of Cleaved-Casp3, BAX and BCL-2 in NRCMs with the corresponding analysis. *P < 0.05 compared with NC; ^#^P < 0.05 compared with HF + HG; Data are means ± SD

After the primary cardiomyocytes were stimulated by high fatty acid (HF) and high glucose (HG), the level of apoptosis was significantly increased, and the ratio of BAX/BCL-2, Cleaved-Caspase3/GAPDH increased. The ratio of BAX/BCL-2 and Cleaved-Caspase3/GAPDH was significantly decreased after finerenone treatment in NRCMs (Fig. 3C). TUNEL test showed that under the stimulation of HF and HG, the proportion of TUNEL positive cells increased, and the proportion of positive cells decreased after finerenone treatment (Fig. 3B). At the same time, we also performed Annexin V/PI staining and detected by flow cytometry. Compared with the control group, after HF and HG stimulation, the proportion of cells in the early stage of apoptosis (Annexin V+) and late stage of apoptosis (Annexin V+/PI+) increased, and the overall percentage of apoptotic cells increased (Fig. 3A). After treatment of finerenone, the proportion of early apoptosis and late apoptosis decreased, suggesting that finerenone has a protective effect on apoptosis induced by HF and HG stimulation.

### Transcriptome data suggest finerenone improves cardiomyocytes in multiple ways

**Fig. 4:**
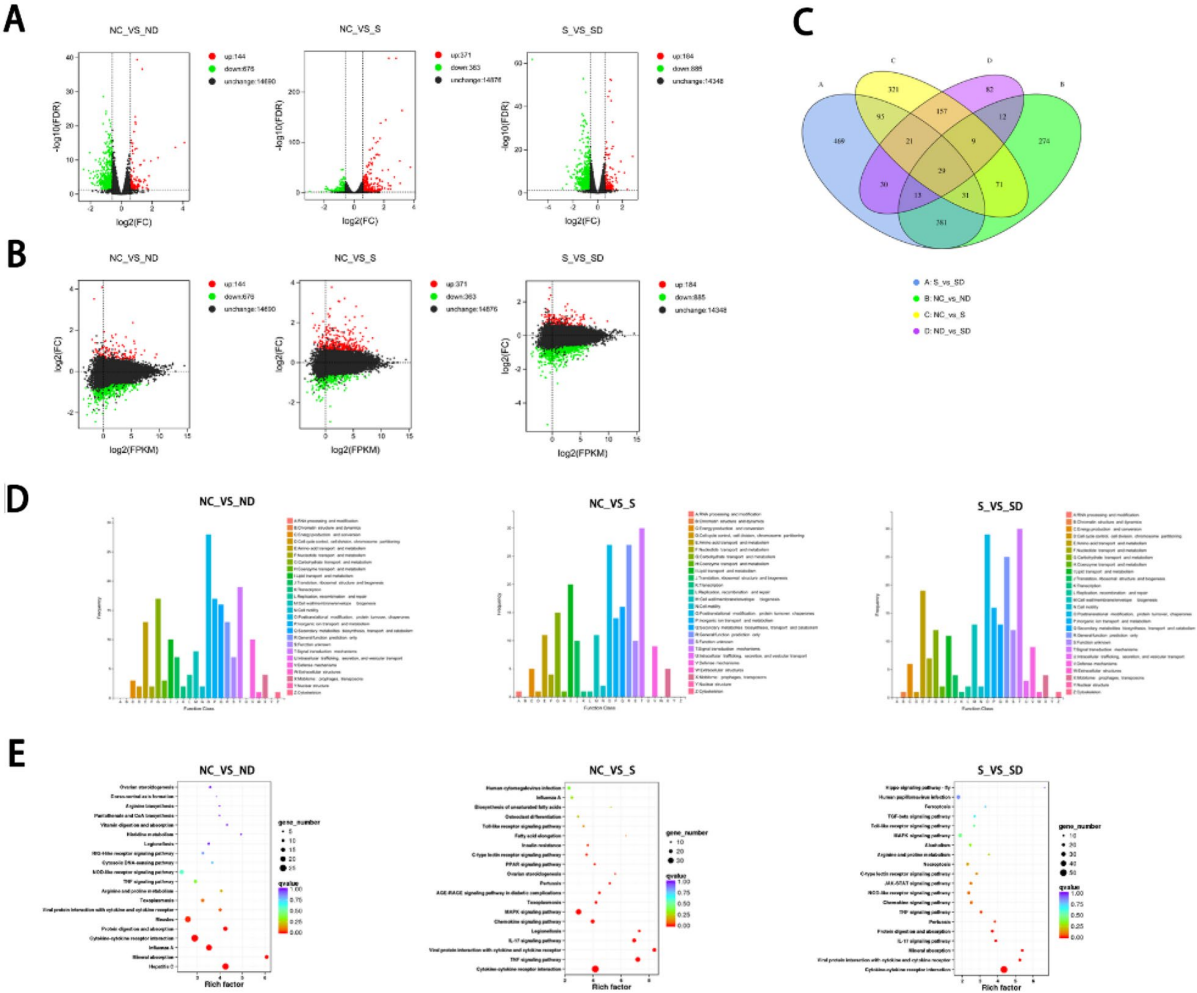
Transcriptome results reveal that finerenone ameliorates high glucose and high fatty acid-induced inflammation and metabolic abnormalities in neonatal rat cardiomyocytes. **A** The volcano plots. **B** The MA plot. **C** The Venn diagram. **D** COG Function Classification of Consensus Sequence. **E** KEGG pathway enrichment analysis. Gene function was annotated based on the following databases: Nr (NCBI non-redundant protein sequences); Pfam (Protein family); KOG/COG (Clusters of Orthologous Groups of proteins); Swiss-Prot (A manually annotated and reviewed protein sequence database); KO (KEGG Ortholog database); GO (Gene Ontology). NC (negative control group), S (HF + HG stimulation group), ND (negative control with drug group), SD (HF + HG stimulation with drug group)

By RNA-seq and transcriptome analysis, 734 genes were altered between control (Group NC) and HF + HG stimulated (Group S) cells under differential conditions (set fold change > 1.5, P-value < 0.05). Compared with the HF + HG group treated with finerenone (Group SD), a total of 1069 genes were changed, 184 genes were up-regulated, and 885 genes were down-regulated (Fig. 4A–C). The COG database was used to classify and count the differential genes. Compared with the control group, after HF + HG stimulation, the signal transduction mechanism, post-translational modification, lipid transport and metabolism and other genes were changed. Compared with the experimental group, the changed genes were concentrated in signal transduction mechanism, post-translational modification, amino acid transport and metabolism, lipid transport and metabolism in the treatment group (Fig. 4D). KEGG pathway enrichment analysis of differential genes showed that the inflammatory signaling pathways TNFα, IL-17, etc. were up-regulated in the HF + HG stimulation group compared with the control group, and these pathways were all down-regulated in the treatment group (Fig. 4E). Taken together, these data suggest that high glucose and high fatty acid stimulation can cause changes in a series of genes related to inflammation and metabolism, and finerenone alleviates these changes.

### Finerenone improves cardiomyocyte metabolism and reduces ROS generation through PPARγ/CD36 pathway

**Fig. 5:**
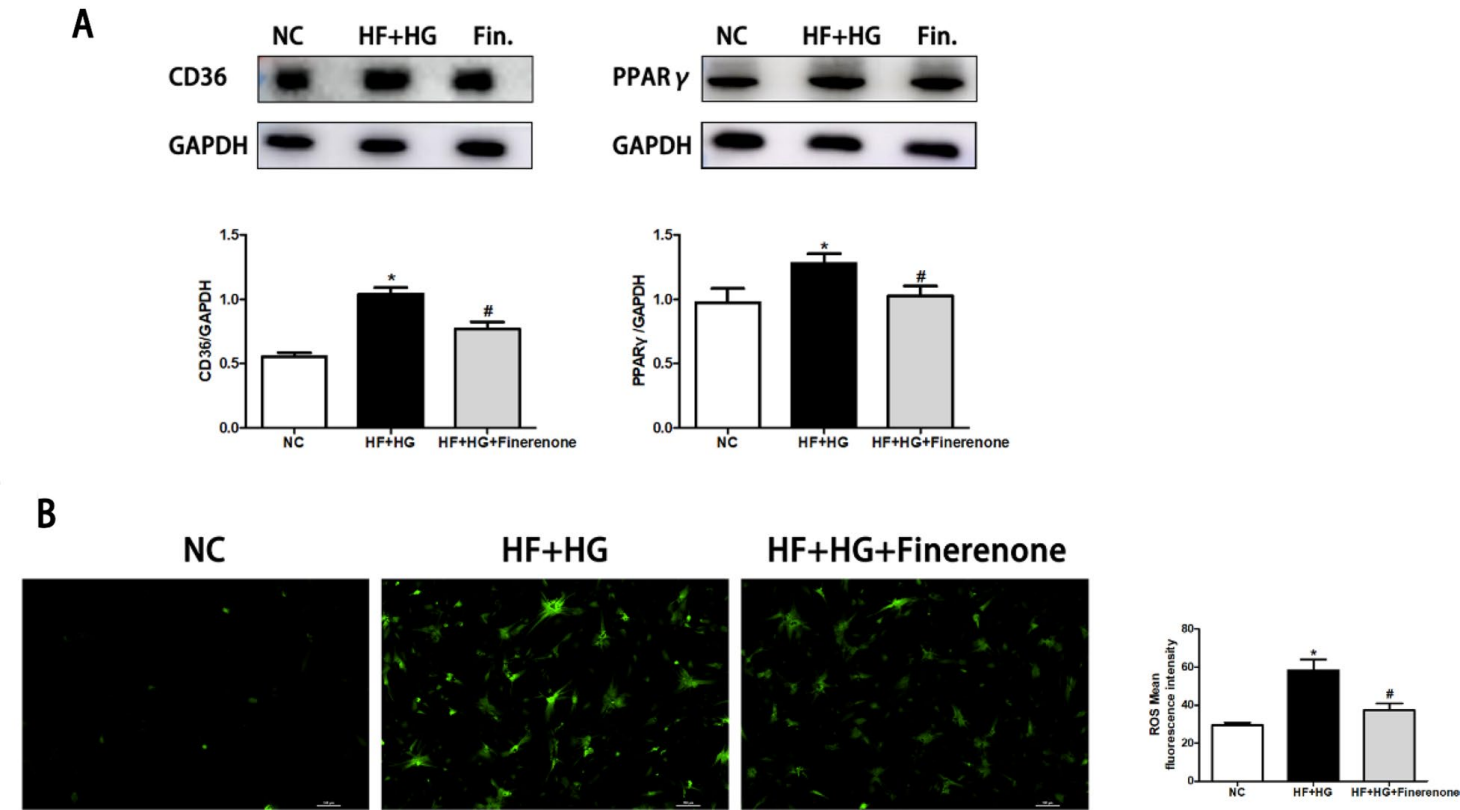
Finerenone reduces PPARγ and CD36 expression and reduces ROS generation under high glucose and high fatty acid stimulation in neonatal rat cardiomyocytes. **A** Representative images of the Western blot of Cleaved-Casp3, BAX and BCL-2 in NRCMs with the corresponding analysis. **B** The production of ROS with the corresponding analysis. *P < 0.05 compared with NC; ^#^P < 0.05 compared with HF + HG; Data are means ± SD

Cardiomyocyte apoptosis in DCM may be caused by excessive uptake and accumulation of FFA. The major FFA protein transporter is CD36 [18]. Under the stimulation of high glucose and high fatty acid, the expression of PPARγ and CD36 increased, and the expression of PPARγ and CD36 decreased after finerenone treatment (Fig. 5A). At the same time, after the cells take in excess lipids, the formation of ROS will be increased [19]. Compared with the control group, the ROS in cardiomyocytes was significantly increased under high glucose and high fatty acid stimulation, and the ROS decreased significantly in the finerenone treatment group (Fig. 5B). This suggests that finerenone may improve cardiomyocyte metabolism through the PPARγ/CD36 pathway.

### Finerenone reduces apoptosis in neonatal rat cardiomyocytes stimulated by high glucose and high fatty acid via TNFα/TNFR1 pathway

**Fig. 6:**
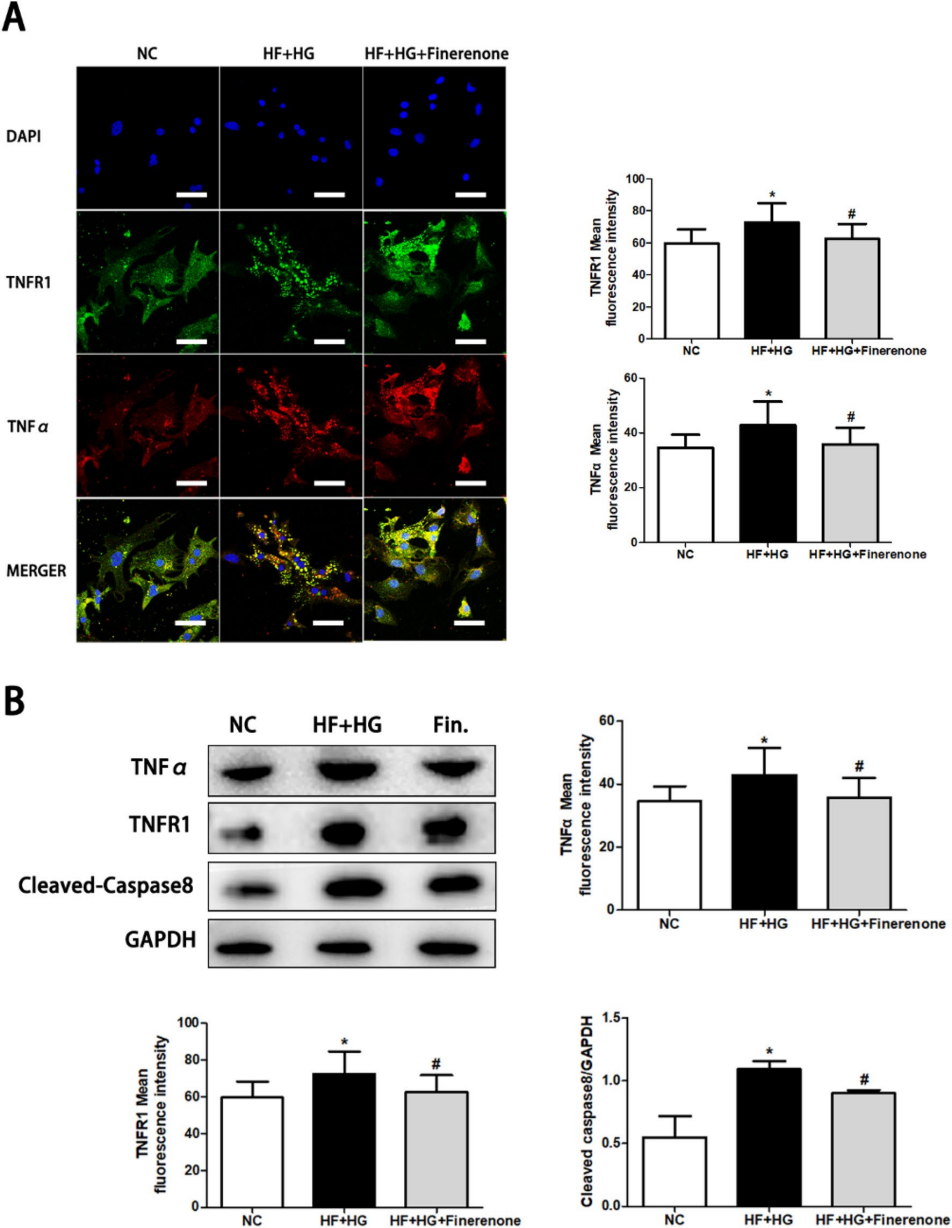
Finerenone reduces apoptosis in neonatal rat cardiomyocytes stimulated by high glucose and high fatty acid via TNFα/TNFR1 pathway. **A** Confocal images of TNFA and TNFR1 with the corresponding analysis. **B** Representative images of the Western blot of TNFα, TNFR1 and Cleaved-Caspase8 in NRCMs with the corresponding analysis. *P < 0.05 compared with NC; ^#^P < 0.05 compared with HF + HG; Data are means ± SD

The transcriptome suggested that, compared with the control group, the TNFα pathway was up-regulated in the high-glucose and high-fat stimulation group, and the TNFα signaling pathway was down-regulated after finerenone treatment. We localized TNFα and TNFR1 by confocal microscope, and found that the expression of TNFα and TNFR1 in cardiomyocytes increased under high glucose and high fatty acid stimulation, and the expression of both decreased after finerenone treatment (Fig. 6A). By western blot, compared with the control group, the expression of TNFα, TNFR1, and Cleaved-Caspase8 in the high glucose and high fatty acid group was up-regulated, and the up-regulation of these proteins was alleviated after treatment with finerenone (Fig. 6B). This indicates that finerenone relieves cardiomyocyte apoptosis under high glucose and high fat stimulation via the TNFα/TNFR1/CASPASE8 pathway.

## Discussion

DCM is an important complication of diabetes, which was first proposed by Rubler et al. in 1972 [3]. After controlling for other risk factors, compared with individuals without diabetes, men with diabetes had a 2.4-fold increased risk of heart failure and women a five-fold increase. Elevated lipid content in the myocardium, increased apoptosis and ECM deposition are characteristic manifestations of DCM [4].

Aldosterone, a mineralocorticoid steroid hormone produced in the glomerular zone of the adrenal cortex, stimulates mineralocorticoid receptors in the heart in addition to its regulatory effects on the kidneys [20]. Since Thorne et al. [21] proved that DOCA-treated dogs had sodium chloride retention, people gradually studied and understood aldosterone. Soon, it was demonstrated that MR activation was associated with inflammation and fibrosis in various organs [22]. The first-generation MRA spironolactone and the second-generation MRA eplerenone were gradually discovered and synthesized, and showed good cardiac benefits [23–25]. However, the application of these MRAs has significant limitations due to the properties of steroid hormone receptors. Steroid hormone receptors include the subfamily of nuclear receptors acting as intracellular receptors and nuclear transcription factors, which is composed of androgen receptors, glucocorticoid receptors, mineralocorticoid receptors, progesterone receptors, and estrogen receptors. Together, their structural similarities make MRA prone to non-specific activation of other receptors [26]. For example, spironolactone is prone to cause breast pain and gynecomastia [24], which is related to its higher affinity with estrogen receptors. However, eplerenone may also limit its application in cardiac therapy because of the risk of hyperkalemia [27, 28]. In 2013, finerenone was discovered, which is highly specific to MR [10]. In the following large-scale clinical trials, finerenone showed good cardiac benefits [12, 29], causing. It is of interest to us to study its specific mechanism.

In this study, we found that the structure and function of the heart improved in diabetic rats treated with finerenone. Based on the results of histological stain and western blot, we found that finerenone can reduce cardiac fibrosis and myocardial apoptosis. In order to further study its mechanism of action, we sent the cardiomyocytes to transcriptome analysis after being stimulated with high glucose and high fatty acids and drugs. Transcriptome results suggested that finerenone affected signal transduction mechanisms, post-translational modifications, amino acid transport and metabolism, lipid transport and metabolism. We analyzed the relevant results and decided to conduct further research on lipid metabolism and signal transduction.

Excessive accumulation of lipids can damage cardiac function [28]. PPARy is a transcriptional regulator of genes that encode proteins involved in lipid metabolism [30]. Studies showed that PPARγ in diabetic myocardium is significantly up-regulated [31, 32], and excessive activation of PPARy in myocardium can cause damage to myocardium [31]. In our study, after treatment with finerenone, PPARγ/CD36 in cardiomyocytes under high glucose and high fatty acid stimulation was down-regulated, suggesting that finerenone may regulate fatty acids intake in cardiomyocytes through the PPARγ/CD36 pathway.

Under the stimulation of high glucose and high fatty acid, the induced cell apoptosis is related to the increase of intracellular ROS. Under the stimulation of high glucose and high fatty acid, the increase in lipid uptake by cells can lead to an elevation in ROS levels, thereby inducing cell apoptosis [18]. In addition, we also found that inflammatory pathways, such as TNFa pathway and IL-8 pathway, were up-regulated under the stimulation of high glucose and high fatty acid. Among them, the TNFα/TNFR1 signaling pathway activates Caspase 8 through the FADD domain, and then activates Caspase3, which also increases the apoptosis of cardiomyocytes [33]. Through confocal and western blot experiments, we verified that finerenone can reduce the apoptosis of cardiomyocytes by down-regulating the TNFα/TNFR1/CASPASE8 pathway.

## Conclusion

**Fig. 7:**
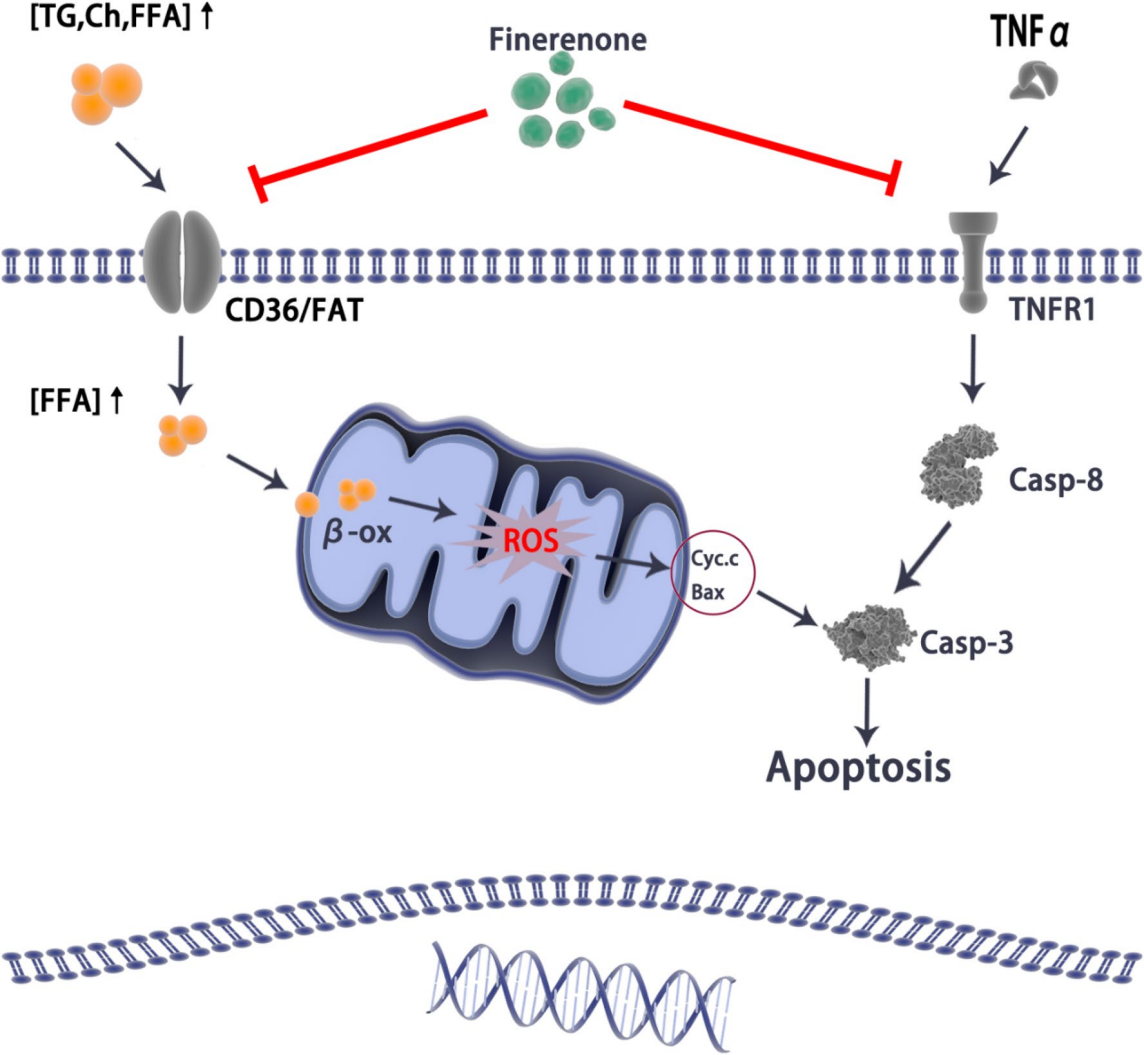
Potential protective effects of finerenone in T2DM hearts. Finerenone down-regulates the expression of CD36 on the cell membrane surface, thereby reducing lipid uptake and ROS generation. At the same time, finerenone can regulate the TNFα/TNFR1/CASPASE8 pathway, reduce the apoptosis of cardiomyocytes, and protect the myocardium

In summary, the protection of finerenone on the heart is multifaceted. It can improve lipid metabolism in cardiomyocytes and reduce myocardial lipid uptake by down-regulating PPARγ/CD36. It can also down-regulate the TNFa/TNFR1/CASPASE8 signaling pathway to reduce the apoptosis of cardiomyocytes (Fig. 7). Through this experiment, we proposed the molecular mechanism of finerenone to protect the myocardium, hoping to provide finerenone with more possibilities for clinical application.

## Supplementary Information

**Additional file 1. **Western blot.

## Data Availability

The datasets used and analyzed during the current study are available from the corresponding author on reasonable request.

## Abbreviations

TNFα: Tumor necrosis factor alpha
PPAR: Peroxisome proliferators-activated receptors
DCM: Diabetic cardiomyopathy
FFA: Free fatty acids
FA: Fatty acids
ROS: Reactive oxygen species
RAAS: Renin–angiotensin–aldosterone-system
MRAs: Mineralocorticoid receptor antagonists
MR: Mineralocorticoid receptor
IPGTT: Intraperitoneal glucose tolerance test
IPITT: Intraperitoneal insulin tolerance test
FBG: Fasting blood glucose
LVEF: Left ventricular ejection fraction
FS: Fractional shortening
HE: Haematoxylin and eosin
NRCMS: Neonatal rat cardiomyocytes
DMEM: Dulbecco’s modified eagle medium
BSA: Bovine serum albumin
DCFH‐DA: Dichlorofluorescein‐diacetate
SDS-PAGE: Sodium dodecyl sulphate polyacrylamide gel electrophoresis
SD: Standard deviation
T2DM: Type 2 diabetes mellitus
TC: Total cholesterol
HDL-C: High-density lipoprotein cholesterol
LDL-C: Low-density lipoprotein cholesterol
TG: Total triglyceride
GLU: Glucose
ALD: Aldosterone
HW: Heart weight
NC: Negative control
HF + HG: High fatty acid and high glucose
